# Distributed nestmate recognition in ants

**DOI:** 10.1098/rspb.2014.2838

**Published:** 2015-05-07

**Authors:** Fernando Esponda, Deborah M. Gordon

**Affiliations:** 1 Department of Computer Science, Instituto Tecnológico Autónomo de México, México D.F. 01080, Mexico; 2 Department of Biology, Stanford University, CA 94305, USA

**Keywords:** nestmate recognition, natural computing, distributed systems, cuticular hydrocarbons

## Abstract

We propose a distributed model of nestmate recognition, analogous to the one used by the vertebrate immune system, in which colony response results from the diverse reactions of many ants. The model describes how individual behaviour produces colony response to non-nestmates. No single ant knows the odour identity of the colony. Instead, colony identity is defined collectively by all the ants in the colony. Each ant responds to the odour of other ants by reference to its own unique decision boundary, which is a result of its experience of encounters with other ants. Each ant thus recognizes a particular set of chemical profiles as being those of non-nestmates. This model predicts, as experimental results have shown, that the outcome of behavioural assays is likely to be variable, that it depends on the number of ants tested, that response to non-nestmates changes over time and that it changes in response to the experience of individual ants. A distributed system allows a colony to identify non-nestmates without requiring that all individuals have the same complete information and helps to facilitate the tracking of changes in cuticular hydrocarbon profiles, because only a subset of ants must respond to provide an adequate response.

## 1. Introduction

Distributed processes are widespread in nature as well as in engineered data networks [1]. In systems without central control, the role of an individual part depends on its interactions with other individuals. For example, in the developing fly brain, lateral inhibition among neighbouring cells regulates which cells eventually differentiate to become the sensory bristles [2]. Another example is the vertebrate immune system, in which there is no single cell that is able to identify every possible antigen; instead individual cells can each detect a small subset [3]. Because an antigen is encountered by a variety of immune cells when it enters the organism, there is a high probability that it will be identified, thus conferring thorough coverage by the aggregate. The immune system can track changes in ‘self’ efficiently because only a few immune cells must be updated for every change. This provides a robust security system, difficult to subvert since there is no single cell where all the information about ‘self’ is kept and from which it can be stolen, hijacked or copied [4]. Methods for data privacy and computer security [5–7], like the immune system [8], rely on fragmenting and distributing sensitive information among a set of agents so that each one holds only a piece of the puzzle. Their collective characteristics confer adequate coverage for the host organism; together they define what is acceptable by individually specifying what is not.

Distributed processes regulate not only the collective behaviour of cells, but also that of groups of organisms [8]. Here, we propose a model of nestmate recognition in social insects in which recognition depends on a distributed, colony-wide process. Nestmate recognition has been observed in many species of social insects [9], including wasps [10,11] and bees [12–15]. Our model focuses on ants but may be broadly applicable to other taxa. Ants are an extremely diverse and widespread taxon of more than 14 000 species. All ant species live in colonies, and nestmate recognition is crucial in regulating colony cohesion and interactions with other colonies. Ants are ecologically important in every terrestrial ecosystem, and competition among colonies has important effects on ant population dynamics (e.g. [16]). In many species, colonies engage in ongoing interactions with neighbouring colonies that are crucial for the partitioning of resources [17–19].

Many studies of nestmate recognition show considerable variation among individuals in the extent of aggression (e.g. [13,20,21]). Studies of nestmate recognition in ants rely on behavioural assays, with individuals from different colonies placed in proximity, to determine when one ant identifies another as belonging to a different colony. Recognition is considered to occur if individuals are more aggressive towards non-nestmates than towards nestmates. The results of such studies are notoriously variable (e.g. [20,22–24]). The outcomes depend on whether the experiments were conducted blind [24], on the worker task [23,25] and on the numbers of ants included in the assay [20]. Often individuals of the same colony differ in response (e.g. [26]).

The variability among individuals in their aggressive response to individuals from other colonies has been treated as a methodological problem rather than as a fundamental feature of the process of nestmate recognition. The variability is probably owing to many factors, such as variation among individuals in their ability to detect chemical profiles [27] and in differences among task groups in the likelihood that they will respond in a bioassay [23]. However, current understanding of nestmate recognition is built on the premise that variation among individuals of the same colony in response to another colony is mostly owing to noise or undetected influences of social context [28].

The model for nestmate recognition presented here is based on a process in which individuals recognize non-nestmates based on previous experience, with the consequence that individuals within a colony differ in recognition response. Nestmate recognition by the colony is achieved through a distributed process, so that overall, ants from one colony distinguish those from another, but individuals do not always do so. We argue that variation makes the system more effective and accounts for results that have until now been taken to be owing to noise.

Our model of nestmate recognition begins with the premise that individuals acquire experience about nestmate and non-nestmate odours over time. Ants learn odour cues through exposure [26,29–32], over the course of hours [33,34] and even as larvae [35], so that different ants may react differently to the same chemical profile, and an individual ant may slowly change its reaction to a given odour. Odour recognition in ants appears to be inclusive; ants seem to be better at recognizing the presence of a new compound rather than its absence [36,37]. Here, we consider how nestmate recognition can emerge from the distribution of various learned odours among ants within a colony.

## 2. Distributed detection model

**Figure 1.**
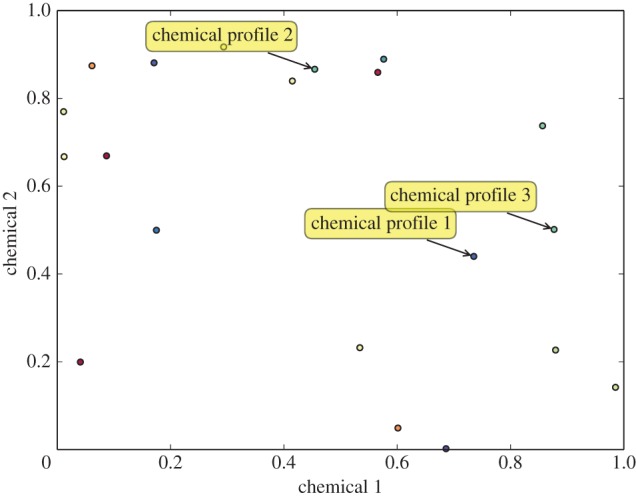
CHC-space for two chemicals. Each point represents a particular combination of the chemicals 1 and 2.

Nestmate recognition in social insects is olfactory. Hydrocarbons present on an insect's cuticule (CHC) are sensed by other workers and used to discriminate between nestmates (individuals from the same colony) and non-nestmates (those from other colonies). We describe the relation among chemical profiles as in [38] with reference to cuticular hydrocarbon or CHC-space, an *n*-dimensional Euclidean vector space in which each coordinate axis represents the concentration of a given hydrocarbon (figure 1). A particular chemical profile is a point in this multi-dimensional space. We assume that when an ant interacts with another one, for example when two ants engage in antennal contact, the focal ant can evaluate the absolute quantities of various components in the other ant's CHC profile [39] and estimate the position of the other ant's hydrocarbon profile in CHC-space.

The notion of CHC-space has been used in many studies to refer to the similarity between templates or profiles. For example, the template similarity model [40] describes recognition as a function of the distance between an individual's internal template, corresponding to the colony-specific CHC profile of that individual's colony, and the cues that individual detects from other individuals. In Newey's [38] model, recognition depends on the position, in CHC-space, of an encountered odour relative to a region defined by the distance between the individual's innate odour and that of its colony. In the D-present and U-absent model [41], workers use distances along axes, defined by specific desirable or undesirable cues, to accept or reject individuals. In all of these models, the colony odour is known to be a simplification; individuals are known to differ slightly in CHC profile. As Van Zweden *et al.* [42] point out, the results of these models depend on how the colony odour is defined.

**Figure 2.**
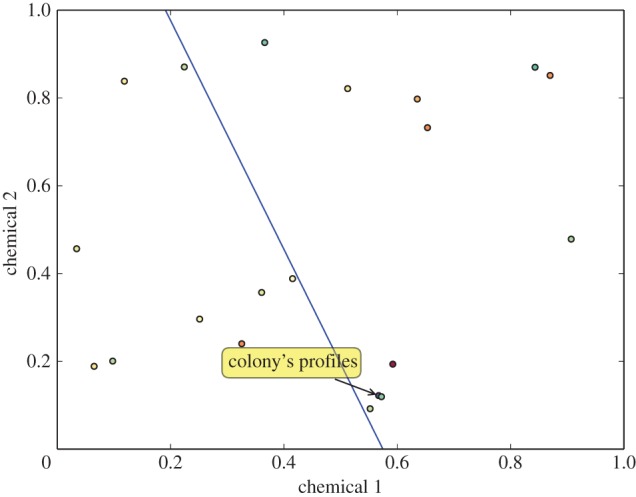
A single ant's linear decision boundary in CHC*-*space. Any odour profiles on the other side of the colony's profiles are considered by the ant to be non-nestmates.

**Figure 3.**
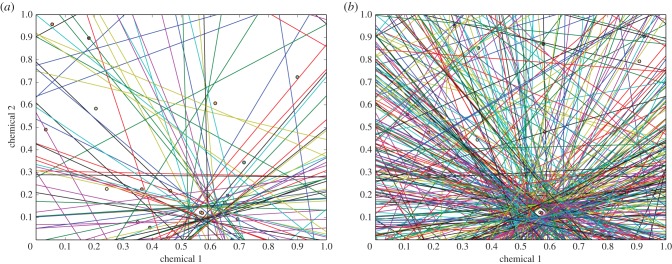
Overall colony recognition. Each line represents a boundary in CHC-space for one individual. The region of odour profiles that will be accepted by the colony, corresponding to the region on the nestmate side of the boundary for all individuals, is located in the small open area in the lower, central part of the figure, at about coordinates [0.58, 0.10]. The figures are based on the logistic regression model and habituation algorithm of §2a. Panels (*a*) 100 and (*b*) 500 separating boundaries.

Here we propose that each ant carries, and can update, the description of a boundary that partitions CHC-space into two subsets: nestmate and non-nestmate. Each ant's boundary is the result of its individual history of encounters with both nestmates and non-nestmates (figure 2). Because ants differ in experience, each ant's boundary is different, and no individual ant's boundary is the same as that of the colony as a whole. Instead, the diverse positions of this boundary for all ants intersect to create the colony's profile in CHC-space. This colony template in turn determines the colony's response to non-nestmates (figure 3). In the figures we represent this boundary as a line to illustrate the idea as simply as possible, but the shape of this boundary in the many-dimensional CHC-space could itself vary among ants within a colony, and is influenced by the mechanisms involved in perception.

The figures were created using the logistic regression model described in §2a; each boundary is initialized with a random set of weights and habituated to a set of patterns representing the colony's profile.

We assume that each ant is consistent in its perception of a chemical profile over the timescale under consideration, and that the ant classifies all odours from a particular other colony in the same way.

Let *p_i,j_* be the proportion of individuals from colony *i* that recognize *j* as a non-nestmate chemical profile. Then2.1
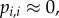
2.2
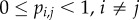
2.3
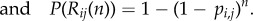
Equation (2.1) states that nestmate profiles tend to elicit no agonistic response, although in large colonies, when there are many nestmates that never meet, it may be that occasionally an ant recognizes a nestmate as a non-nestmate. This is probably rare since it has almost never been observed. Equation (2.2) states that each ant can recognize only a subset of possible non-nestmate chemical profiles. Equation (2.3) describes the probability of the recognition event *R* in which an ant of colony *j* is recognized as a non-nestmate by at least one out of *n* individuals from colony *i,* and models our assumption that each ant recognizes a different and independent subset of chemical profiles, so that the colony's nestmate recognition is distributed. Because individuals tend to meet particular others of certain chemical profiles, for example nestmates of the same task group, who may tend to share chemical profiles [43], it is unlikely that the boundaries of ants are completely independent, but we use this assumption to simplify our model. Equation (2.3) describes an equilibrium that occurs on the timescale over which each ant is consistent in its perception of a chemical profile and consistently recognizes a unique subset of non-nestmate odours.

Finally, as a corollary of equation (2.3), the probability that a recognition event occurs between *n* ants of colony *i* and *m* ants of colony *j* is2.4
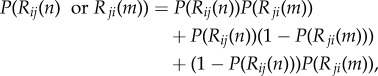
where the last two terms refer to unilateral recognition.

In the following, we use aggression as a proxy for recognition, since aggression implies recognition (although we recognize that the reverse is not true). The probability of aggression is a fraction of the probability of *R*2.5
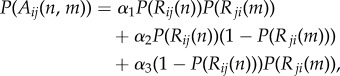
where the *α_i_*s establish the probability of aggression for bilateral and unilateral recognition.

We now consider how an ant's boundary between nestmates and non-nestmates in CHC-space might shift over time. Such shifts would lead to continual changes in the colony's response to non-nestmates.

### (a) Shift in individual recognition

An ant's boundary between nestmates and non-nestmates in CHC-space can be understood as the chemical combinations for which there are sufficient neural activity to trigger perception of a non-nestmate. We suggest that boundaries shift as a result of a process similar to the one reported in studies such as those of Guerrieri *et al.* [44] and van Zweden & d'Ettorre [9], in which response to nestmate odours is reduced by exposure to nestmates. The boundary is established by habituation, and continually modified by experience, for example by repeated encounters with an unfriendly conspecific non-nestmate.

As an example we describe how CHC profiles could be learned using the collective action of a metaphorical set of neurons referred to as the ‘recognizer’, modelled as a logistic regression [45]. Each recognizer receives the information corresponding to *m* inputs or cues and has a weight associated with each one that determines its importance. The recognizer fires, producing an output according to: 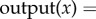 
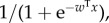
 where *x* is the input pattern vector whose components are individual hydrocarbons, and *w* is a weight vector of real numbers that express the positive or negative role of each hydrocarbon. The output function makes rapid and continuous transitions between 0 and 1. We model each ant as having an internal threshold beyond which recognition of a non-nestmate takes place:
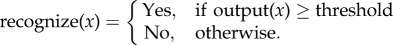


The exact value for the threshold is not important, so without loss of generality, assuming a threshold = 0.5, the boundary that separates recognition as non-nestmate from recognition as nestmate is when (1/(1+e^0^))=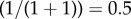, which occurs when *w*^T^ x* = 0. These are the values for *x* plotted in figures 2 and 3. Each ant's separating boundary between nestmates and non-nestmates arises from its particular set of weights *w*.

Learning in this context is the process that finds suitable values for *w*. Diversity among ants arises because ants differ in weights. We assume that over successive encounters, ants habituate to an odour if each encounter with the odour is not associated with aggression, or if there is a positive experience such as trophallaxis or grooming [9,23,34,44,46], while aggression or the absence of a positive experience leads to gradual sensitization to non-nestmate odours. This assumption is supported by previous studies. Knaden *et al.* [47] and Van Wilgenburg *et al.* [48] found that in successive encounters between non-nestmates, aggression increased. An exception to this was reported by Nowbahari [49], who found that ants of *Cataglyphis niger* reduced aggression towards particular individual non-nestmates in successive encounters, suggesting that an ant may habituate to the odour of a particular non-nestmate. However, in successive encounters with different non-nestmate individuals, aggression did not decrease.

Weights could be adjusted by a procedure akin to the Delta rule [50] whereby small changes to the positive and negative weights are made in the appropriate direction in response to each encounter. In this way, a simple mechanism, providing each ant with a crude characterization of chemical profiles, can collectively distinguish nestmates and non-nestmates (figure 3) for the colony as a whole. Although ants probably use much richer neural processes to perform this task [33,51], a simple mechanism is sufficient to model recognition behaviour.

## 3. Discussion

### (a) Fit with existing data

In this section, we consider how results from the literature support our model. We focus primarily on studies that track the response of individual workers towards non-nestmates in repeated encounters.

Experiments by Newey *et al.* [26] on weaver ants (*Oecophylla smaragdina* L.) show that in repeated exposures, an individual ant reacted similarly to similar chemical profiles, but different ants from the same colony responded differently to the same chemical profile. A further experiment, in which an individual from the recipient colony met workers from different colonies, showed that a particular ant reacts differently to different odours. These findings support our assumptions that workers have a boundary that consistently separates the same nestmate and non-nestmate odours, that not all non-nestmate odours are perceived as such by a given individual, and that individual workers within a colony differ in the boundary that separates nestmate from non-nestmate chemical profiles.

Newey *et al.* [26] suggest that there is a set of diverse templates based on individual odours and that recognition requires both an individual and a colony profile, with aggressive response as a function of the distance between the two [38]. Their explanation is that each ant has encoded a different tolerance area in CHC-space defined by both the individual's innate odour and the colony odour. In our model, by contrast, the region of rejection for an individual is not bounded by its own odour; thus, although individuals differ in profile, an ant with a profile closer to that of the colony may exhibit the same aggressive behaviour as an ant whose profile is further from that of the colony. This occurred in a study of harvester ants (*Pogonomyrmex barbatus*) [23]; other studies are needed to determine whether this occurs in many ant species and under what ecological conditions.

Roulston *et al.* [20] investigated how aggressive behaviour between non-nestmates depends on the number of ants in each trial. Their experiments report the proportion of trials that have at least 1 aggressive encounter in assays with 1 versus 1, 1 versus 25, and 5 versus 5 live ants, as well as an assay with 1 live versus 1 dead ant. In a distributed model such as ours and Newey's [38], different ants identify a different subset of chemical profiles so, for assays with few ants (1 versus 1), we expect high variability among replicates as was observed, and the average over many replicates to approximate the real frequency of aggression in 1 versus 1 encounters. The distributed model would also explain why a more consistent aggression score was observed in the 5 versus 5 assays, since it includes several 1 versus 1 interactions.

Our model predicts that the probability of aggression in encounters between non-nestmates depends more on the number of different non-nestmates that each ant encounters, than on the total number of ants or total number of interactions. For example, aggression is more likely when 5 ants of colony A meet 5 ants of colony B, than when 1 ant of colony A meets 25 ants of colony B. In the first case, there are 5 ants that might recognize B as a non-nestmate, while in the second case, only 1 ant may recognize B, and if it does not recognize one B it is unlikely to recognize the other 24. We tested this quantitatively using the data from [20] and equation (2.3), which establishes the probability that a foreign ant is recognized as a function of the number of encounters, and equation (2.5) with *α*_1_ = 1 and *α*_2_ = *α*_3_ = 0. We assume only two-sided recognition because there was less aggression in the live versus dead than live versus live assays in Roulston *et al.* [20], and other work [23] suggests that aggression is more likely when both participants recognize the other as non-nestmates. This produces numbers lower than the observed experimental averages, because one-sided aggression does sometimes occur. We approximate *p_i,j,_,* the proportion of ants of colony *i* that recognize the colony profiles of colony *j* as that of non-nestmates in each experiment, using equation (2.5) by taking the square root of the observed proportion of aggressive encounters in the 1 versus 1 assays for each pairing. We thus take *p^2^_i,j_* to reflect the probability of an aggressive act between two ants. Our predictions, along with the results of Roulston *et al.* [20], are shown in electronic supplementary material, table S1. Our model yields a good approximation for the observed ordering in the frequency of aggression for the 1 versus 1, 1 versus 25 and 5 versus 5 experiments.

Although there were an equal number of possible encounters in the 1 versus 25 experiments and in the 5 versus 5 assays, the level of aggression was lower (electronic supplementary material, table S1), as expected, since we assume that a particular individual classifies all odours from the same colony in the same way: in terms of equation (2.3), *P*(*R_i,j_*(1)) = 1 − (1 − *p_i,j_*) = *p_i_*. Conversely, each of the 1 ant's 25 adversaries has an independent chance of recognizing the single ant; using equation (2.3), *P*(*R_j,i_*(25)) = 1 − (1 − *p_j,i_*)^25^. Thus the probability of observing an aggressive encounter in the 1 versus 25 assay using equation (2.5) is *P*(*A_i,j_*(1,25)) = *P*(*R_i,j_*(1)) *P*(*R_j,i_*(25)). In the 5 versus 5 assays, each single ant classifies all the odours from its adversary colony in the same way, but in contrast with the 1 versus 25 assay, both colonies have many (five) individuals in the match. Using equation (2.3), *P*(*R_j,i_*(5)) = *P*(*R_i,j_*(5)) = 1 − (1 − *p_j,i_*)^5^ so the probability of aggression is *P*(*A_i,j_*(5,5)) = *P*(*R_i,j_*(5)) *P*(*R_j,i_*(5)). Because we assume that a single ant is consistent in its classification of a colony's odours and each ant recognizes as foreign a distinct subset of chemical profiles, for a given value of *p_i,j_*, *P*(*A_ij_*(1,1)) *≤ P*(*A_ij_*(1,25)) *≤ P*(*A*_*ij*_(5,5)). This result is consistent with the observations (electronic supplementary material, table S1). There were exceptions in two trials, which our model does not account for: in FORb-FORs and EMI-GRF.

Next we consider how the results in Van Wilgenburg *et al.* [48] support our model's assumption that ants show consistent differences in which chemical profiles they recognize. Pairs of ants from different colonies were put together, and later, one ant from each pairing, the focal ant, was placed with another ant of the same colony as the non-nestmate from the previous trial. About 52% of the initial assays resulted in aggressive behaviour, and 78% of the focal ants showed the same behaviour, aggressive or not, during their second encounter with a different non-nestmate of the same colony. Results did not differ when the focal ants were presented with non-nestmates of other colonies; however, non-nestmate CHC profiles may have converged in response to a similar laboratory diet [44]. These results suggest that ants of one colony differ consistently in their sensitivity to particular non-nestmate CHC profiles.

During the second encounter, the percentages of trials with aggressive behaviour for the initially passive and initially aggressive ants were 29% and 89%, respectively, leading to an overall increase in aggressive behaviour from 52% in the first round of encounters to 60% in the second round. There are several alternative explanations for the observed increase. If recognition of a non-nestmate odour is random, so that an individual is equally likely to recognize a given odour as nestmate or non-nestmate in consecutive encounters, then the expected distribution of passive and aggressive ants during the second round would be the same as in the first round. According to this explanation, the observed distributions in [48] would have to be owing to two distinct unexplained shifts: a moderate (19%) diminution in aggression for the initially passive ants, from 48 to 29%, and a large (37%) increase in aggression for the initially aggressive, from 52 to 89%.

Another possibility is that the passive ants remain passive and the aggressive ants remain aggressive, leading to 0% aggression for the initially passive ants and 100% aggression for the initially aggressive set. Then the observed distributions would have to be produced by two unexplained shifts: a large increase in aggression for the initially passive ants (29%) and a moderate decrease in aggression (11%) for those that were initially aggressive. This interpretation is consistent with the authors' hypothesis that exposure to an adversary, even in the absence of a fight, increases the likelihood of subsequent aggression.

An alternative explanation provided by our model is that the observed distributions are owing only to one shift: a moderate increase in the aggression (17%) of the initially aggressive ants. In our model, each ant is consistent, during a short time period, in recognizing particular odours as those of non-nestmates, and again we assume that aggression is more likely between two non-nestmates if both individuals recognize each other as non-nestmates than if only one recognizes the other as a non-nestmate (as in [23]). When we then calculate the expected distributions of passive and aggressive ants during the second round, assuming no overall increase in aggression (electronic supplementary material, appendix S2), we obtain 30% and 72% of aggressive encounters for the initially passive and initially aggressive ants, respectively, in contrast to the observed 29% and 89%. We suggest that the observed increase in aggression is primarily explained by a heightened propensity for aggression, even in the absence of bilateral recognition, for the initially aggressive ants (as reported by Hsu *et al.* [52] in other taxa). In contrast to the experiments in [49], which showed a diminution of aggression between particular ants in successive encounters, ants in this study [48] did not face the same adversary in the second round. Thus, our model provides a simple explanation for the observed results that requires only a single process. Further experiments are needed to test this explanation.

Guerrieri *et al.* [44] demonstrate that the addition of certain components to an ant's CHC profile make it susceptible to aggression by its untreated nestmates. This is consistent with our predictions. Treated ants were left together to acclimate, providing opportunities for repeated encounters, before the bioassays were performed. Aggression by a group of untreated ants towards a treated one was higher than that by a group of treated ants towards an untreated one. Our model suggests that the boundaries of the treated ants adjusted to include the added compound, so that treated ants were likely to recognize the untreated ants as nestmates, because the odour of the untreated ants was included in the treatment mixture. However, an untreated ant would detect as foreign the presence of an added compound if its decision boundary were close to that of the colony's odours. Such asymmetry in recognition was reported by Bos *et al.* [36], and the mechanism they propose might account for the results of Guerrieri *et al.* [44].

In another study, Sturgis & Gordon [23] demonstrated that the probability of aggression depends on task group: exterior workers are significantly more aggressive than interior workers, and recognition is more likely towards ants of neighbouring colonies [29,53], which are often encountered by exterior workers [18], than ants of more distant colonies. These results suggest that aggression is related to the past history of encounters: individuals that have previously met non-nestmates are more likely to recognize them [47,48].

Honeybee guards outside the hive react towards intruders that are non-nestmates. However, recognition of non-nestmates by bees tends to be inaccurate, with a mean rejection rate of non-nestmates of only 51% (mean based on Johnson *et al.* [21]). This is consistent with our model: individual bees vary in the boundary in CHC-space that separates the odours of nestmates and non-nestmates and that recognition is distributed. Other studies show that in the short-term, individuals are consistent in which odours they recognize as non-nestmates [12,54], and habituation to conspecific odours has been demonstrated in several studies [54–56] suggesting that a bee's recognition ability changes as a result of its interactions with other bees.

Our model may help to explain the apparently contradictory results on neighbour recognition in ants. Some studies show that ant colonies distinguish the odour of neighbouring colonies [29,30,57]; for example, encounters with neighbouring foragers inhibit foraging more than encounters with ants from distant ones. This is puzzling because field and laboratory studies show that it is unlikely for any individual ant to meet others of the same neighbouring colony many times [58,59]. We suggest this effect may be owing to small shifts in the recognition boundary of ants who have met a non-nestmate only a few times. Consider two neighbouring colonies, A and B, and a third colony C, so distant that ants from A and B never encounter ants of C. Every encounter that an ant from A has with an ant from B shifts that A ant's boundary slightly. However, repeated encounters between A and B do not systematically affect the relationship between A and C, whose ants never meet. In a test such as the one in [29], in which large numbers of ants from A can encounter some from B, some ants from A are likely to respond to B, because some of them have previously met an ant from B, but none are as likely to respond to C, because none of the A ants have ever met any C ants. Whether such recognition results in aggression or a ‘dear enemy’ response [30,57] may depend on the particular ecological conditions that determine the extent of competition and costs of aggression between neighbours.

### (b) Predictions and conclusions

We propose a distributed model of nestmate recognition that predicts the variability in ant aggressive behaviour that is consistently observed in experimental assays. Our model differs from other models of nestmate recognition in describing recognition as a function of the position of a detected odour with respect to a boundary surface in CHC-space, rather than as a function of the distance between odours (figure 2). Our proposal integrates features of other models: it supports Guerrieri *et al.*'s [44] conclusion that ants recognize non-nestmates rather than nestmates, and generalizes Newey's [38] proposal by allowing arbitrary shapes for the colony decision boundary in CHC-space that are not bound by the innate odours of particular individuals. Our model also extends the D-present, U-absent [41] and U-present [9,44] mechanisms by modelling an individual as perceiving a chemical bouquet in which some cues can be excitatory and some inhibitory. In contrast to previous models, in our model each ant may be sensitive to different cues, for example by detecting odour as a linear combination of chemicals containing both positively and negatively weighted cues. Thus, our model posits a malleable decision boundary for the colony that emerges from the aggregated experiences of each individual. The decision boundary could arise from a variety of mechanisms, such as the pre-filter [60,61] or neural template [34,62] mechanisms, which may vary among species.

We suggest that each ant in a colony learns to recognize a unique set of non-nestmate odours, so that ants are likely to agree more in accepting nestmates than in aggression towards non-nestmates. Our model is similar to that of Johnson *et al.* [21], in that each ant has a limited recognition capability, and recognition of non-nestmates is the result of the collective reaction of the colony, but differs strongly from theirs in its assumptions and thus in its predictions (see [63] for comments on this model). In Johnson *et al.*'s [21] simulation, individual recognition ability is random, while in ours, recognition ability is a consequence of prior experience [26,29–32,44] and thus consistent in response to specific odours at any given time. We suggest that ants hold a neurologically encoded decision rule that takes the form of a separating surface in CHC-space (§2a). Thus identity is not inscribed in a template commonly shared by all ants in a nest; it is partially encoded in each of its members and distributed among them (figure 3).

Further work is needed to test the predictions of our model. First, it predicts that individuals that initially do not respond aggressively towards a particular odour can develop an aggressive response towards that odour if it is associated with negative experience such as aggression. This has been found in several studies, including Knaden & Wehner [47] and Van Wilgenburg *et al.* [48]. More tests of this are needed.

Second, we predict that the more an individual is exposed to non-nestmates, the greater the precision of its discrimination of non-nestmates. In general, aggressive behaviour should be more likely, the more non-nestmates an ant has encountered. Repeated encounters with diverse odours will tend to position the boundary closer, on average, to the set of odours of its nestmates, i.e. to the colony profile. The closer an ant's boundary to the colony profile, the broader the range of non-nestmate chemical profiles that it will be able to recognize, and the more accurately it will be able to identify foreign odours. This can be tested with experiments involving repeated encounters, such as those in Van Wilgenburg *et al.* [48], to test whether, as an ant is exposed to more other ants, it can identify as non-nestmates ants whose chemical profile is more similar to its own because its boundary between nestmates and non-nestmates moves closer to its own colony's profile (figure 3). This prediction is supported by the widespread observation that recognition mistakes in the form of aggression between nestmates are extremely rare, while mistakes in the form of a lack of aggression, or failure to recognize non-nestmates, are extremely common. It appears that the initial response, or default response of a young ant, is lack of aggression. A bias towards lack of aggression towards nestmates, because the vast majority of a worker's encounters are with nestmates, favours colony cohesion at the risk of allowing intrusion or other harm from non-nestmates. Younger ants tend to work inside the nest [64], where they are likely to meet only nestmates. The more similar are the odours of nestmates, the more likely they are to be on the same side of the boundary. Only later, as they work outside the nest, e.g. as foragers, are they likely to meet ants of other colonies. Thus, as some studies show [23], ants that leave the nest, such as foragers, should be more likely to respond aggressively to non-nestmates than ants that work only inside the nest, such as brood care workers.

Third, we predict that although in general, the distances between chemical profiles in CHC-space should be correlated with the probability of aggression [9], recognition is not a simple function of the distance between an individual's innate odour and the colony's profile. Instead it is a function of the distance between an ant's decision boundary and the intruder's expressed odour. Thus, two odours may be very close to each other but on distinct sides of the decision boundary, while another two may be far from each other yet classified together, depending on where they lie relative to the ant's boundary.

This implies that the relationship between the probability of recognition and the distance between profiles in CHC-space should change as a result of individual experience, as shown in [36,44,65]. Our model predicts, in contrast to the two-odour model [38], that an individual could accept, as a nestmate, another one with a profile that is actually further than its own from the overall colony's profiles. Studies consistent with this prediction include those of Fielde [66], with colonies formed of mixed species combined after eclosion, and the recent result that in harvester ants, CHC profiles of some task groups were more similar within task group, across colonies, than within colonies [23]. Further studies such as those of [46] that measure individual odour in isolated ants are needed to investigate this.

Evidence against our model would come from studies that establish a lack of variation among individuals in response to odours. An example would be the observation that every ant in a colony identifies the same set of non-nestmates, and that they vary only in their amount of aggression. This could be addressed using a neurophysiological measure, rather than aggression, to determine whether recognition occurs. Other evidence against our model would be the demonstration that aggression is random with respect to odour, without any effect of previous exposure. However, there seems to be substantial evidence that this is not the case.

Using a distributed model of nestmate recognition allows colonies to take advantage of large numbers of individuals to devote fewer resources to the identification of non-nestmates. Colony hydrocarbon profiles change over time [67,68]. A distributed system facilitates tracking of these changes, because only a subset of ants must respond to the change to provide an adequate response. The apparent variability in experimental results in studies of recognition by ants reflects not noise, but an effective method for maintaining colony security. As in distributed systems elsewhere in nature, such as in the immune system, distributed detection allows colonies to adapt to slow changes in colony odours, allows ants to share the energetic burden of detection and provides the colony with a robust security system. The system favours lack of aggression towards nestmates, risking possible errors in the recognition of non-nestmates, in the same way that the immune system favours the inhibition of autoimmune responses while risking infection. A distributed process for recognition may be important in providing group identity to aggregates in many natural systems.

## Supplementary Material

Table S1

## Supplementary Material

Appendix S1
