# Supplementary material for: Distributed nestmate recognition in ants
Source: Proc Biol Sci. 2015 May 7;282(1806):20142838. doi: 10.1098/rspb.2014.2838 (PMC4426612; doi:10.1098/rspb.2014.2838)
Supplement: Table S1 [file rspb20142838supp1.docx]

Table S1: Results from (20) are shown in plain text; results predicted from our model are shown in italic. Each number represents the proportion of trials in which aggression occurred for assays involving: 1-dead: 1 ant paired with a dead non-nestmate, 1-1: one ant from each colony; 1-25: 1 ant from one colony and 25 from the other; 5-5: 5 ants from each colony. *p_i,j_* is the estimated recognition probability used to compute the predicted values, calculated as the square root of the observed 1-1 aggressive encounters (see Eq. 4).
