# Supplementary material for: Distributed nestmate recognition in ants
Source: Proc Biol Sci. 2015 May 7;282(1806):20142838. doi: 10.1098/rspb.2014.2838 (PMC4426612; doi:10.1098/rspb.2014.2838)
Supplement: Appendix S1 [file rspb20142838supp2.docx]

Appendix 1.

Estimate, based on our model, of the results of experiments in Wilgenburg et al. (48 ).

We estimate as follows that 20% of the focal ants that did not display aggression in the first round did recognize their adversaries as non-nestmates but were not themselves recognized as such. The proportion of ants that recognize the other ant as a non-nestmate is the square root of the observed 1 vs 1 aggression rate (Eq. 5 with α_1_=1 and α_2_=α_3_=0): *p_i,j_* =$\sqrt{0.52}$=0.72. Since 52% of ants showed aggression in the first round, implying recognition, an additional 20% (0.52+0.20=0.72) of ants recognized the chemical profile but did not engage in aggression because they were not recognized by the other ant. This 20% comprises 42% (0.20/0.48=0.42) of the initially passive ants. The ants then faced a fresh set of adversaries during the second round, some of which recognized the adversaries' chemical profile as foreign; therefore, the expected percentage of aggressive encounters during the second round for the initially passive ants is *P(A_i,j_(1,1))=*42%*72%=30%, where 72% is the proportion of fresh adversaries that recognize the focal ant’s chemical profile and 42% is the proportion of the focal ants that were initially passive, because they were not recognized, but that do recognize their adversaries. The expected proportion of aggressive encounters in this subset is 30%, in close agreement with the observed 29%. On the other hand, all of the initially aggressive focal ants recognize their adversary's odor as foreign during the second round, while 72% of the adversaries, as calculated above, will recognize the focal ant as foreign. We thus expect to see a 72% level of aggression, *P(A_i,j_(1,1))=*100%*72%, for these ants during the second round.
